# Sp1 and Sp3 Are the Transcription Activators of Human *ek1* Promoter in TSA-Treated Human Colon Carcinoma Cells

**DOI:** 10.1371/journal.pone.0147886

**Published:** 2016-01-25

**Authors:** Chee Sian Kuan, Wei Cun See Too, Ling Ling Few

**Affiliations:** School of Health Sciences, Health Campus, Universiti Sains Malaysia, 16150 Kubang Kerian, Kelantan, Malaysia; University of Saarland Medical School, GERMANY

## Abstract

**Background:**

Ethanolamine kinase (EK) catalyzes the phosphorylation of ethanolamine, the first step in the CDP-ethanolamine pathway for the biosynthesis of phosphatidylethanolamine (PE). Human EK exists as EK1, EK2α and EK2β isoforms, encoded by two separate genes, named *ek1* and *ek2*. EK activity is stimulated by carcinogens and oncogenes, suggesting the involvement of EK in carcinogenesis. Currently, little is known about EK transcriptional regulation by endogenous or exogenous signals, and the *ek* gene promoter has never been studied.

**Methodology/Principal Findings:**

In this report, we mapped the important regulatory regions in the human *ek1* promoter. 5’ deletion analysis and site-directed mutagenesis identified a Sp site at position (-40/-31) that was essential for the basal transcription of this gene. Treatment of HCT116 cells with trichostatin A (TSA), a histone deacetylase inhibitor, significantly upregulated the *ek1* promoter activity through the Sp(-40/-31) site and increased the endogenous expression of *ek1*. Chromatin immunoprecipitation assay revealed that TSA increased the binding of Sp1, Sp3 and RNA polymerase II to the *ek1* promoter in HCT116 cells. The effect of TSA on *ek1* promoter activity was cell-line specific as TSA treatment did not affect *ek1* promoter activity in HepG2 cells.

**Conclusion/Significance:**

In conclusion, we showed that Sp1 and Sp3 are not only essential for the basal transcription of the *ek1* gene, their accessibility to the target site on the *ek1* promoter is regulated by histone protein modification in a cell line dependent manner.

## Introduction

Phosphatidylethanolamine (PE) is the second most abundant phospholipid in eukaryotic cells [1]. PE can be synthesized by CDP-ethanolamine pathway (Kennedy pathway) and phosphatidylserine decarboxylase (PSD) pathway but the former is known to contribute to the majority of the PE in mammalian cells [2]. Ethanolamine kinase (EK) is the first enzyme in the CDP-ethanolamine pathway, which catalyzes the phosphorylation of ethanolamine by using ATP to yield phosphoethanolamine and ADP. In human, EK exists as three isoforms, encoded by two separate genes, named *ek1* [NCBI Gene ID: 55500] and *ek2* [NCBI Gene ID: 55224]. While *ek1* codes for a single protein, EK1 (452 amino acids), *ek2* undergoes alternative splicing to produce two other EK isoforms, EK2α (386 amino acids) and EK2β (394 amino acids) [3].

High EK activity has been previously shown to correlate with cell growth [4–8]. Induction of EK activity by oncogenes and carcinogens suggested the involvement of EK in carcinogenesis by promoting cell growth and/or survival [4–6]. Malewicz *et al*. [8] showed that overexpression of EK in NIH 3T3 fibroblasts protects fibroblast cells from apoptotic cell death. Increased phosphorylation of ethanolamine and its analogs (methylethanolamine and dimethylethanolamine) in EK overexpressor cells increases cell survival in serum-free medium but decreases the insulin-dependent DNA synthesis [8].

Our preliminary experiments showed that the *ek1* promoter activity was significantly induced under serum starvation condition. Serum starvation induced genes transcription through the modulation of chromatin structure by core histones modifications [9–12]. Acetylation of lysine residues on histone tail “relaxed” the packed chromatin and provides accessibility to transcription machinery that leads to transcriptional activation. Conversely, histone deacetylases (HDACs) catalyze the deacetylation of lysine residues on core histone, allowing compacted chromatin structure to form and repress transcription [13]. Inhibition of HDACs by trichostatin A (TSA) induced the expression of CTP: phosphocholine cytidylyltransferase α (*Ctpctα*), the second enzyme in the CDP-choline pathway, through the Sp1 binding site of its promoter [14]. The expression of choline kinase α (*ckα*), the first enzyme in the CDP-choline pathway, was also stimulated by treatment of MCF-7 cells with HDAC inhibitor, suberoylanilide hydroxamic acid (SAHA) [15].

Although EK is the key enzyme in cellular phospholipid synthesis and it has been implicated in cell growth and carcinogenesis, the regulation of human *ek* gene at the transcriptional level has never been described. This study aims to characterize human *ek1* promoter by identifying the *cis*-acting element that regulates *ek1* basal expression. Since *ek1* promoter is rich in GC sequences with several Sp-family transcription factor binding sites, the role of Sp proteins in the *ek1* transcription regulation was investigated. The molecular mechanism that underlies the TSA-mediated induction of *ek1* promoter was also studied.

## Materials and Methods

### *In silico* analysis of *ek1* promoter region

The 1966 bp upstream region of *ek1* gene (transcript NM_018638) was analyzed by TFSEARCH [16] and MatInspector 8.0 [17] to identify the putative transcription factor binding sites. CpGIS [18], with default parameters, was used to determine the CpG island on the *ek1* promoter. CpGIS defines CpG island as sequence having an observed/expected ratio > 0.65, length > 500 bp and GC content > 55%.

### Cell culture

Human liver carcinoma HepG2 [ATCC No: HB-8065], human colon carcinoma HCT116 [ATCC No: CCL-247] and human breast adenocarcinoma MCF-7 [ATCC No. HTB-22] cell lines were cultured in high glucose Dulbecco’s modified Eagle’s medium (DMEM) supplemented with 10% (v/v) heat inactivated fetal bovine serum (FBS), 100 U/mL penicillin and 100 μg/mL streptomycin. Cells were incubated at 37°C in a CO_2_ incubator with 95% (v/v) humidified atmosphere and 5% (v/v) CO_2_.

### Construction of the *ek1* promoter-luciferase reporter plasmids

The *ek1* promoter fragment -1966/+1 was amplified by PCR from human genomic DNA (Roche, Germany) using forward primer (*ek1*-1966-5’) and reverse primer (*ek1*-3’) incorporating *Nhe*I and *Bgl*II sites, respectively. PCR product was cloned in frame at the 5’-end of *Xho*I*/Hind*III site of the promoterless firefly luciferase reporter vector pGL4.10[*luc2*] (Promega, USA), yielding pGL4.10-*ek1*(-1966/+1). The 5’-end deletion constructs of pGL4.10-*ek1*(-1183/+1) and pGL4.10-*ek1*(-665/+1) were made by restriction digestion of pGL4.10-*ek1*(-1966/+1) with *Sac*I/*Xma*I and *Kpn*I, respectively. pGL4.10-*ek1*(-229/+1) was prepared by inserting the *Hind*III digested fragment from the pGL4.10-*ek1*(-1966/+1) into the dephosphorylated *Hind*III digested pGL4.10[*luc2*] vector. pGL4.10-*ek1*(-154/+1), pGL4.10-*ek1*(-100/+1) and pGL4.10-*ek1*(-69/+1) deletion constructs were generated by PCR using pGL4.10-*ek1*(-1966/+1) as the template. The PCR products were subcloned into the *Xho*I*/Hind*III site of pGL4.10[*luc2*] vector. Mutations of Sp binding sites were introduced into pGL4.10-*ek1*(-69/+1) by PCR to produce pGL4.10-*ek1*-mut[Sp(-69/-60)], pGL4.10-*ek1*-mut[Sp(-40/-31)] and pGL4.10-*ek1*-mut[Sp1(-69/-60)/(-40/-31)]. The primers used in this study are listed in Table 1. All deletion and mutation constructs generated by PCR approach were verified by DNA sequencing.

**Table 1:** Primers used for generating promoter-luciferase constructs and PCR site-directed mutagenesis.

Name	Sequence 5’ to 3’	Orientation
**Promoter-luciferase constructs**		
*ek1*-1966-5’	CTA GCT AGC TAC ATC CTG GTA GGG TTG GTC C	Forward
*ek1*-154-5’	CTA GCT AGC GTT CCC AGG GAT GGG TGT G	Forward
*ek1*-100-5’	CTA GCT AGC GAG GTC CCA TTG TGA CCG GAG	Forward
*ek1*-69-5’	CTA GCT AGC CCG CCT CGG CAC CCT GAC G	Forward
*ek1*-3’	GGA AGA TCT TGC CGG GGC TGG CCT GAC G	Reverse
**Site-directed mutagenesis**		
*ek1-*mut[Sp(-40/-31)]	CTA GCT AGC CCG CCT CGG CAC CCT GAC GCA GCG CAG GAC C*t*G C*t*C CGC GCG TGA CGC CAG	Forward
*ek1-*mut[Sp(-69/-60)]	CCT GAG CTC GCT AGC CC*tt*CT CGG CAC CCT GAC GCA GCG CAG	Forward
*ek1*-3’	GGA AGA TCT TGC CGG GGC TGG CCT GAC G	Reverse
**Real-time PCR**		
*ek1*-F	AAAGGTTCCTAAGTGATATCCC	Forward
*ek1*-R	GCCAGGTAGTTGTATCCAGA	Reverse
**ChIP assay**		
*ek1*-154-5’	CTA GCT AGC GTT CCC AGG GAT GGG TGT G	Forward
*ek1*-3’	GGA AGA TCT TGC CGG GGC TGG CCT GAC G	Reverse

The mutations introduced into the binding sites are in lower cases and italicized. Underlined nucleotides are the restriction enzyme recognition sites for cloning.

### Luciferase assay

Promoter reporter constructs were transiently transfected into mammalian cancer cells using Lipofectamine 2000 (Invitrogen) according to the manufacturer’s instruction. Briefly, cells were plated in 100 μL of medium/well on a 96-well plate at a density of 1.5 × 10^5^ cells/well for 24 hr. The cells in each well were co-transfected with 200 ng of promoter reporter constructs or pGL4.10[*luc2*] and 2.5 ng of pGL4.73[*hRluc*/SV40] vector (Promega) All firefly luciferase activities were normalized to (divided by) *Renilla* luciferase activity in the same sample to control for differences in transfection efficiency. Forty-eight hours after transfection, cells were harvested and assayed using Dual-Glo Luciferase Reporter Assay System (Promega). The luminescent signals were measured by GloMax^®^ 20/20 Luminometer (Promega, USA). Each of the luciferase assays was performed in triplicate of three independent experiments.

### Trichostatin A (TSA) treatment

The effect of trichostatin A (TSA) on *ek1* promoter activity was determined by treatment of pGL4.10-*ek1*(-69/+1) and pGL4.73[*hRluc/SV40*] co-transfected HCT116 or HepG2 cells with varying concentrations of TSA (0.125, 0.25, 0.5 and 1 μM for HCT116 and 1 μM for HepG2) for 24 hours. The effect of TSA treatment duration was studied by treatment with 1.0 μM of TSA for 6, 12 and 24 hours. DMSO was added to the cells instead of TSA for negative controls. After treatment, the cells were harvested and assayed by Dual-Glo luciferase assay.

### Quantitative real-time PCR of *ek1* mRNA

Total RNA was purified from TSA treated (1 μM for 24 hr) or control HCT116 cells by using the RNeasy Mini Kit (Qiagen, Germany) according to the manufacturer’s instructions. The RNA purity was assessed by determining the ratio of absorbance at 260 nm and 280 nm. The size distribution and integrity of the RNA were checked by 1% (w/v) agarose gel electrophoresis. The RNA bands were visualized by ethidium bromide staining and observed under UV light. The synthesis of cDNA from 1 μg of total RNA was performed by RevertAid^™^ H Minus First Strand cDNA Synthesis kit (MBI Fermentas, USA). The mRNA level of *ek1* gene was measured by quantitative real-time PCR (qPCR) performed on an ABI PRISM 7000 Sequence Detection System (Applied Biosystems, USA). The intron-spanning *ek1* primers are listed in Table 1. Primers for the reference genes (UBC and YWHAZ) were purchased from TATAA Biocenter (Sweden). The use of single reference gene for normalization could lead to relatively large errors [19]. Hence, YWHAZ and UBC were chosen as reference genes based on our previous report [20] that showed the two genes have the highest expression stability among twelve reference genes tested. The PCR efficiencies of both reference genes were higher than 90% [20]. The PCR efficiency of *ek1* was determined in this study by first generating a standard curve from serial dilution of cDNA template followed by calculation according to this formula: PCR amplification efficiency (%) = (10^−1/slope^−1) × 100% [21]. For the quantitative real-time PCR, each reaction was performed in a 25 μL volume containing 12.5 μL Power SYBR Green I Master Mix, 300 nM of each primer and 1 μL of 1:2 diluted cDNA as template. The cycling program was performed according to the default settings of the ABI PRISM 7000 SDS software 1.0 as follows: 2 minutes at 50°C, 10 minutes at 95°C, followed by 40 cycles of 10 seconds at 95°C and 1 minute at 60°C. Melting curve analysis was carried out immediately after the amplification with temperatures ranging from 60 to 95°C in 0.1°C increments to verify the PCR specificity. The comparative *C*t method was used to calculate the relative mRNA level of *ek1* in TSA treated and control cells [22]. The obtained *C*t value for *ek1* was normalized with the geometric mean of *C*t values for UBC and YWHAZ reference genes [20]. The relative fold change in *ek1* mRNA expression level was calculated by the ΔΔ*C*t method [22].

### Chromatin immunoprecipitation (ChIP)

Chromatin immunoprecipitation (ChIP) assay was performed using Pierce Agarose ChIP Kit (Thermo Fisher Scientific, USA) according to the manufacturer’s protocol. HCT116 and HepG2 cells were cultured with or without TSA (1 μM) for 24 hours. The DNA-protein complexes were immunoprecipitated with 8 μg of Sp1 antibody (Millipore, USA), 8 μg of Sp3 antibody (Millipore, USA) and 8 μg of RNA polymerase II (RNA pol II) antibody (Thermo Fisher Scientific, USA). DNA recovered from samples was PCR amplified using *ek1*-154-5’ and *ek1*-3’ primers followed by agarose gel electrophoresis. The intensities of the bands of interest were quantitated with Image J 1.42 program [23]. All the PCR products were gel-purified and sequenced to confirm that the PCR products were *ek1* promoter.

### Statistical analysis

Statistical analysis was performed using the Student’s *t-*test or one-way ANOVA, followed by Tukey Honestly Significant Differences (HSD) *post-hoc* test. The analysis was performed using the PASW Statistics 18, Release Version 18.0.0 (SPSS, Inc., USA). Data were presented as mean ± SEM of at least three independent experiments.

## Results

### Human *ek1* promoter is a TATA-less, CpG island containing promoter

**Fig 1:**
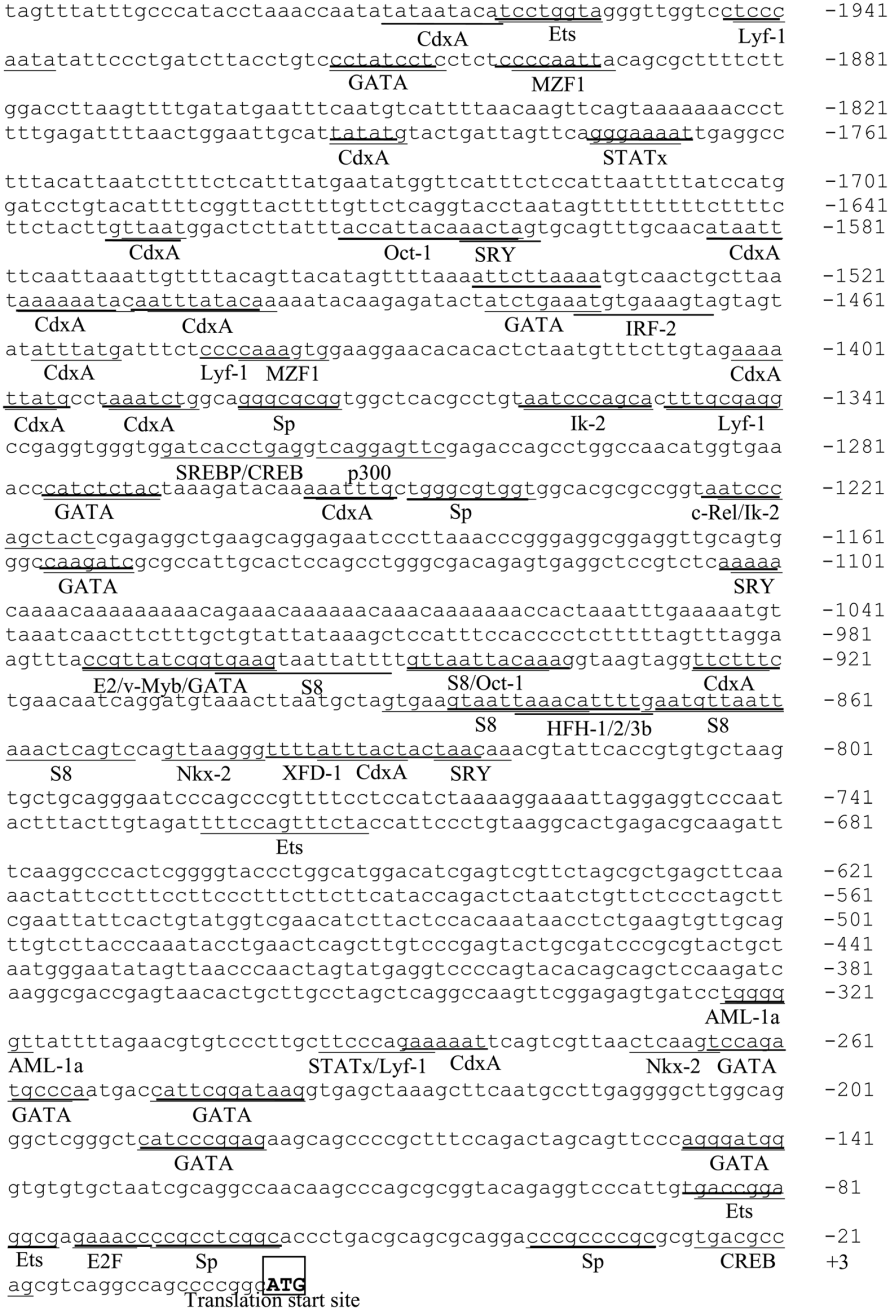
Sequence analysis of *ek1* 5’-flanking region. Underlined sequences are the transcription factor binding sites predicted by TFSEARCH and MatInspector 8.0. Translation start codon ATG is indicated with boldface and boxed.

The sequence of 1967 bp upstream region of the human *ek1* gene (between nucleotides -1966 and +1; +1 refers to the A of the ATG translation start site) was retrieved by combining the database information from NCBI (http://www.ncbi.nlm.nih.gov/) and BLAT server [24]. Analysis of the putative promoter sequence using MatInspector 8.0 and TFSEARCH revealed several potential transcription factor binding sites for Sp-family proteins, Ets, GATA, CREB, MZF1, AML-1a, STATx, Lyf-1, Nkx-2, SRY, Oct-1, S8, and CdxA (Fig 1). The most striking feature of the *ek1* promoter is the lack of a typical CAAT or TATA box, which is a common characteristic of GC-rich promoters. Analysis using CpGIS was thus performed to identify potential CpG island in the *ek1* promoter. The program predicted one CpG island at the region between -529 and -1 with a GC content of 56.3% and an observed/expected ratio of 0.694.

### Activities of *ek1* promoter in HepG2, MCF-7 and HCT116 cells

**Fig 2:**
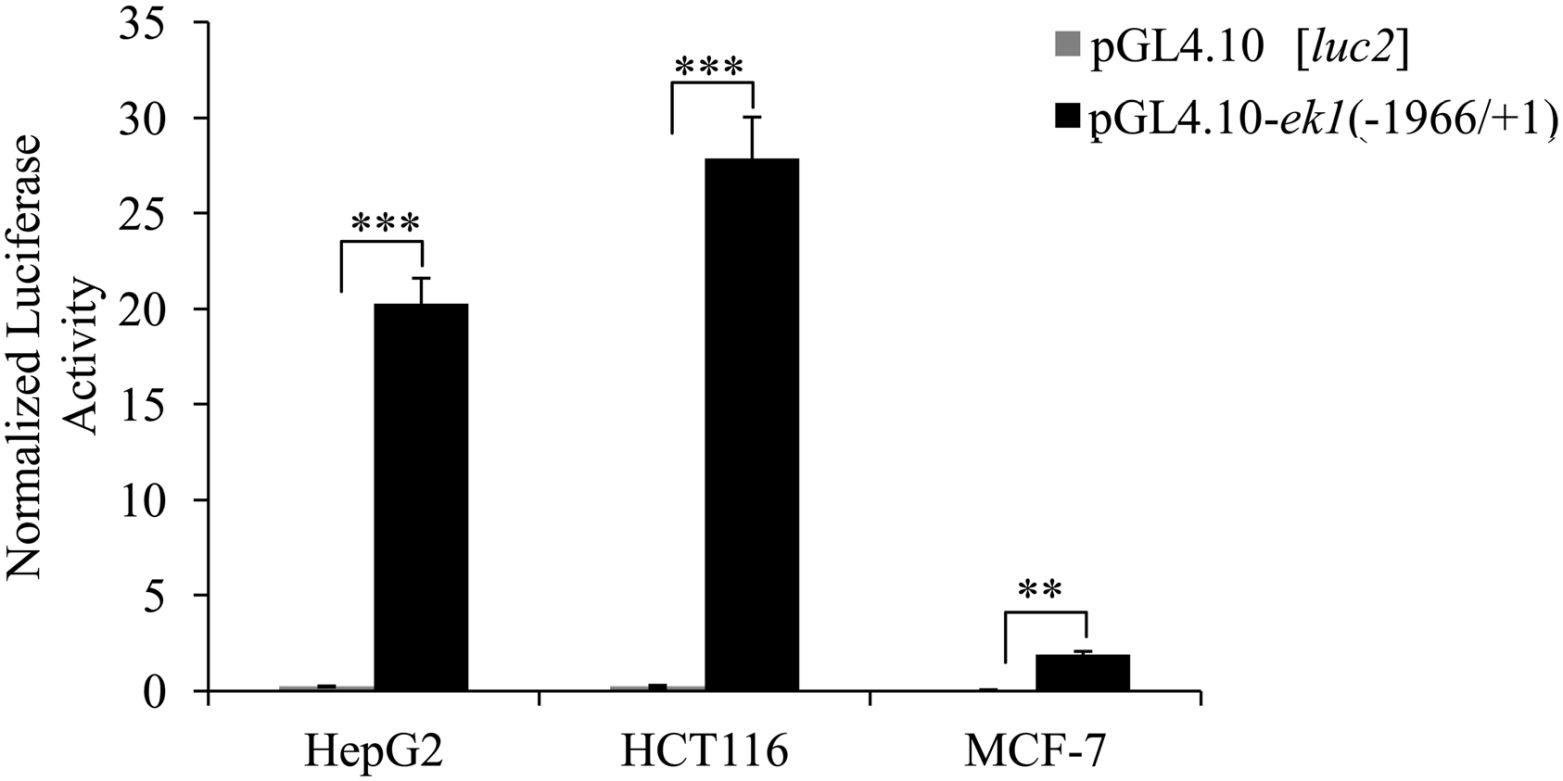
Promoter activities of pGL4.10-*ek1*(-1966/+1) construct in HepG2, HCT116 and MCF-7 cells. Each bar represents the mean ± SEM of triplicate samples from three independent experiments. (** *p* < 0.01; ***p < 0.001 vs. promoterless pGL4.10[*luc2*]).

The functional activity of the pGL4.10-*ek1*(-1966/+1) reporter construct was assessed in HepG2, HCT116 and MCF-7 cells. The cloned *ek1* promoter markedly increased the *luc2* reporter gene expression in all three cell lines as compared to the promoterless control vector (Fig 2). This result showed that the -1966/+1 *ek1* promoter was active in all the cell lines tested with the highest promoter activity detected in HCT116 cells (Fig 2). Hence, HCT116 cells were used as the model cell line for all subsequent *ek1* promoter analysis in this study.

### Identification of important regulatory regions in the *ek1* gene promoter

**Fig 3:**
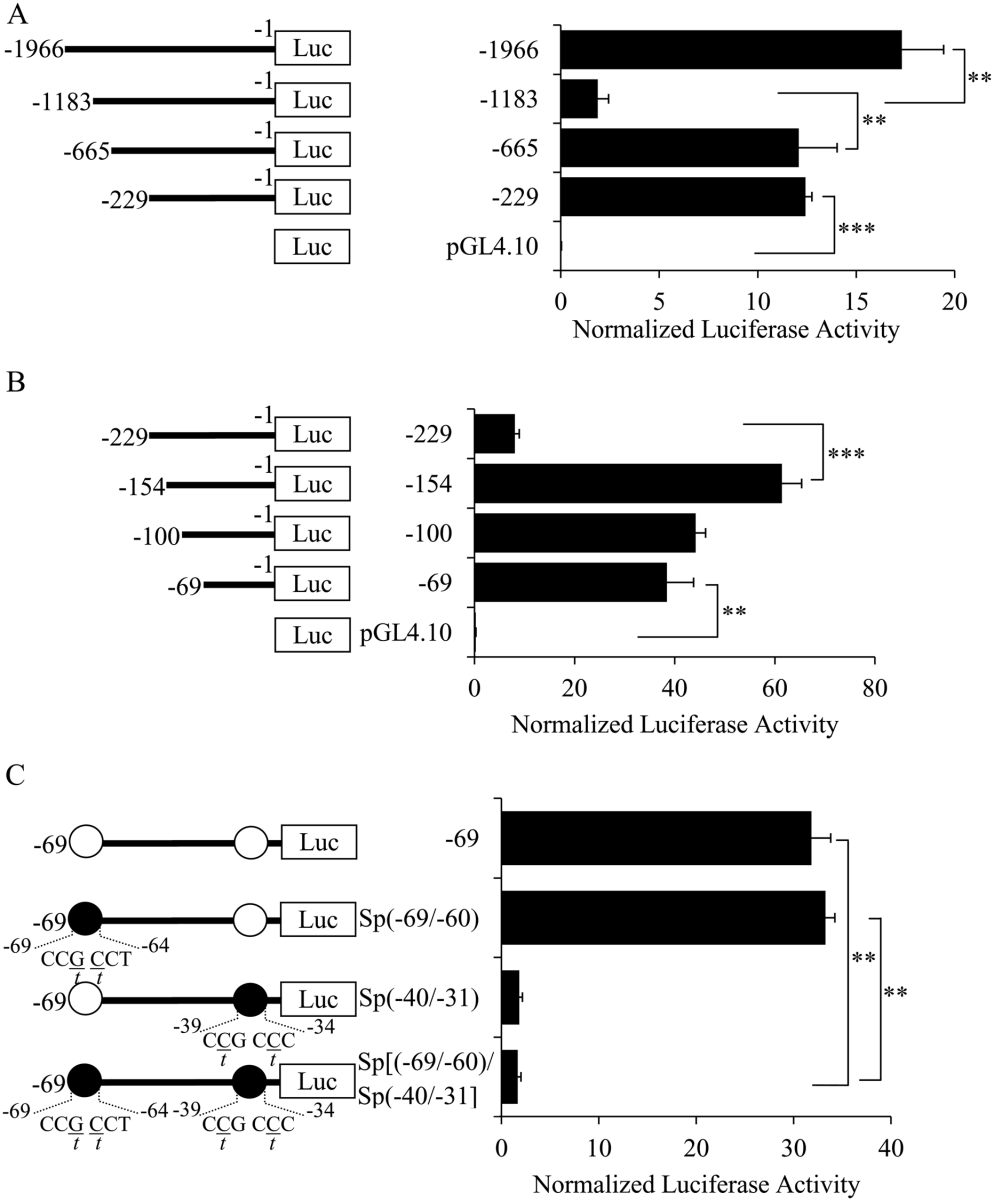
Identification of important region for the *ek1* promoter activity. Promoter activities of luciferase reporter vector containing the *ek1* promoter ranged from (A) -1966 to -229 and (B) -229 to -69. (C) The effect of Sp binding sites mutations in the pGL4.10-*ek1*(-69/+1) reporter construct. Schematic structures of the reporter construct are shown on the left. Binding sites for Sp transcription factors are indicated with open circles. The mutations introduced into the Sp binding sites are underlined and represented as closed circle. Each bar represents the mean ± SEM of triplicate samples from three independent experiments. (**p < 0.01; ***p < 0.001).

The activities of various 5’-deletion constructs of *ek1* promoter were determined to identify the important regulatory regions in this promoter. Fig 3A shows the promoter activities of four constructs [pGL4.10-*ek1*(-1966/+1), pGL4.10-*ek1*(-1183/+1), pGL4.10-*ek1*(-665/+1) and pGL4.10-*ek1*(-229/+1)] produced in the initial round of 5’-deletion analysis. Deletion of the 5’ sequence from the parental promoter construct until the -1182 position resulted in a dramatic decrease of the promoter activity to approximately 11% of the full-length promoter construct. The results suggested that there was at least one positive regulatory element located inside the region between -1966 and -1183. Further deletion of the 5’ end sequence from -1183 to -665 recovered the activity to about 70% of the full length promoter, indicating the presence of at least one inhibitory element in this region (between -1183 and -665). Truncation of the region between -665 to -229 did not cause any significant change of promoter activity. The region downstream of the -229 position was further truncated to map the minimal promoter region for initiating *ek1* gene transcription. As shown in Fig 3B, deletion of the *ek1* promoter from -229 to -154 increased its activity by more than seven times compared to the -229 promoter construct, indicating the presence of at least one repressive element in this region. Subsequent truncations (-100 and -69 promoter constructs) did not show any dramatic change of promoter activity. Thus, the reporter construct containing the -69/+1 *ek1* promoter fragment was taken as the minimal promoter region for conferring basal transcription activity of *ek1*gene.

Bioinformatics prediction of transcription factor binding sites in the -69/+1 *ek1* basal promoter region revealed two putative Sp and one CREB binding sites at positions -69/-60, -40/-31, and -27/-19, respectively. The importance of the two putative Sp binding sites in regulating the *ek1* transcription was examined by single or double mutations of the Sp sites in the pGL4.10-*ek1*(-69/+1) reporter construct. As shown in Fig 3C, single mutation of the Sp(-40/-31) site significantly decreased the promoter activity to approximately 6% of the wild type promoter construct. However, mutation of the Sp(-69/-60) site had no apparent effect on the promoter activity. Double mutation of these two Sp sites significantly decreased the promoter activity to approximately 5% of the wild type promoter construct. The results showed that the Sp(-40/-31) binding site was a functional activator element and it was essential in modulating the basal *ek1* promoter activity.

### TSA activates basal *ek1* promoter activity via Sp(-40/-31) site

**Fig 4:**
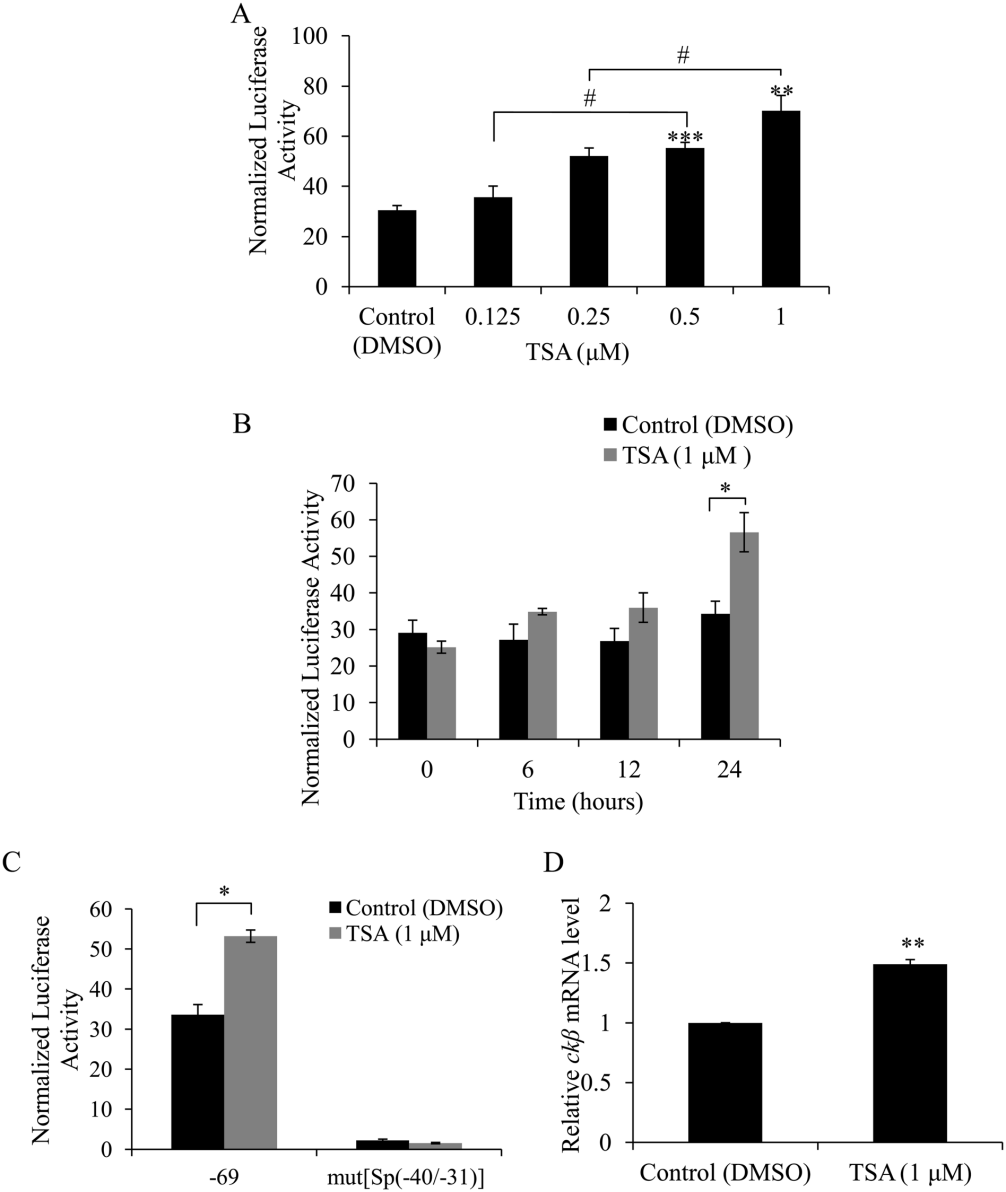
Effect of TSA on the activities of wild type and Sp(-40/-31) mutated *ek1* minimal promoters. A. Promoter activity of pGL4.10-*ek1*(-69/+1) reporter construct in HCT116 cells treated with the indicated concentrations of TSA for 24 hours (*p < 0.001; **p < 0.01; ***p < 0.05 vs. DMSO control; #*p* < 0.05, significant within TSA treatment group). B. Promoter activity of pGL4.10-*ek1*(-69/+1) reporter construct in HCT116 cells treated with 1 μM of TSA for the indicated time points (*p < 0.05 vs. DMSO control). C. Activities of wild type *ek1* minimal promoter and Sp(-40/-31)-mutated *ek1* minimal promoter after treatment with 1 μM of TSA for 24 hours. Each bar represents the mean ± SEM of triplicate samples from three independent experiments. D. Effect of TSA on *ek1* gene expression in HCT116 cells. HCT116 cells were treated with 1 μM of TSA for 24 hours (**p < 0.01 vs. DMSO control). Each bar represents the mean ± SEM of triplicate samples from three independent experiments.

In our preliminary experiments (data not shown), we observed that serum starvation (0.1% FBS) activated the *ek1* promoter and the Sp(-40/-31) site was required for this effect to take place. Since serum starvation could affect genes expression through the modulation of chromatin modification [9, 10], we postulate that the accessibility of transcription factors to the Sp(-40/-31) site responsible for activating *ek1* promoter is modulated by chromatin modification. Histone acetylation/deacetylation is one of the mechanisms to “unravel” chromatin by neutralization of the basic charge of the lysine [25]. To investigate the role of histone acetylation/deacetylation in regulating *ek1* transcription, we treated the pGL4.10-*ek1*(-69/+1) reporter plasmid transfected-HCT116 cells with a well known HDAC inhibitor, trichostatin A (TSA). TSA significantly activated *ek1* promoter in a concentration-dependent manner with the upregulation of the promoter activity started at 0.25 μM and the maximum effect was observed at 1 μM of TSA, which produced 2.3 folds higher promoter activity than the DMSO control (Fig 4A). The effect of TSA treatment duration shown in Fig 4B showed that the *ek1* promoter activity did not significantly increase until 24 hours after TSA treatment with an increase of about 1.6 folds compared to control.

To further confirm the involvement of the Sp(-40/-31) site in the TSA-mediated upregulation of *ek1* promoter, wild type pGL4.10-*ek1*(-69/+1) and Sp(-40/-31) site mutant pGL4.10-*ek1*-mut[Sp(-40/-31)] promoter constructs were transfected into HCT116 cells and treated with 1 μM TSA for 24 hours. As shown in Fig 4C, mutation of the Sp(-40/-31) site completely abolished the TSA-induced *ek1* promoter activation. This result shows that the Sp(-40/-31) site is essential for the activation of *ek1* gene transcription by TSA.

To investigate the effect of HDAC inhibition by TSA on the endogenous expression of the *ek1* gene, the *ek1* mRNA level in the cells treated or untreated with TSA was determined by real-time PCR. All the RNA samples used for cDNA synthesis were intact and pure as indicated by the prominent 28S and 18S ribosomal RNA bands and the ratios of OD_260/280_ from 1.8 to 2.0. The PCR efficiency of *ek1* primers was 89%. Based on the data from triplicate samples from three independent experiments, exposure of HCT116 cells to 1 μM of TSA for 24 hours resulted in moderate but significant increase of *ek1* gene expression by about 0.5 fold compared with the DMSO treated control cells (Fig 4D).

### TSA induced the binding of Sp proteins and RNA polymerase II on *ek1* promoter

**Fig 5:**
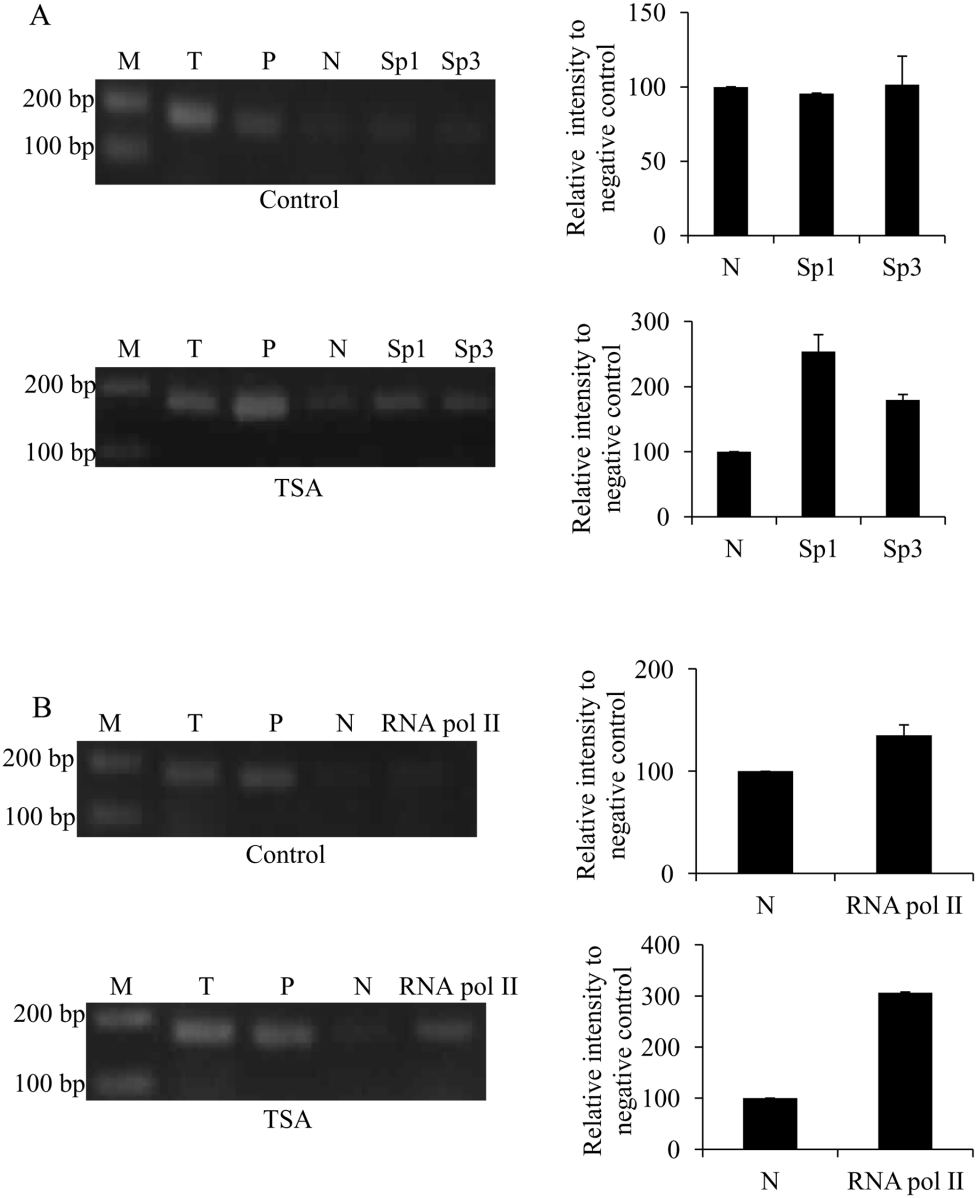
Effects TSA treatment on the binding of Sp1, Sp3 and RNA polymerase II to the *ek1* minimal promoter region. ChIP analysis was performed to confirm the interaction of (A) Sp proteins and (B) RNA polymerase II with the promoter under 1 μM TSA treatment for 24 hours. PCR amplification products were resolved on 2% (w/v) agarose gel and visualized by EtBr staining. Band intensities were quantitated with Image J 1.42 and the relative intensities (compared to negative control) of PCR products from Sp1 and Sp3 immunoprecipitates were plotted. Each bar represents standard error of means (SEM) from two independent experiments. M: GeneRuler^™^ DNA Ladder Mix; T: total input sample (unprocessed chromatin); P: positive control (amplified using GAPDH primers) and N: pre-immune normal rabbit IgG (negative control).

Subsequently, the effect of TSA treatment on the binding of Sp proteins to the Sp(-40/-31) site of *ek1* promoter was also investigated using ChIP assay. In this study, ChIP assay was performed by using Sp1 and Sp3 specific antibodies as these two transcription factors are expressed in the same cells and are indistinguishable in their DNA-binding specificity [26]. The *ek1*-154-5’ and *ek1*-3’ primers used for PCR of the immunoprecipitated DNA are flanking the Sp(-40/-31) response element. The result showed in Fig 5A shows that TSA treatment for 24 hours increased the binding of both Sp1 and Sp3 on the GC-rich *ek1* minimal promoter as compared to control.

The inhibition of HDACs activities by TSA treatment has also been reported to enhance the recruitment of RNA polymerase II to certain promoters [27–29]. Therefore, the effect of TSA treatment on the binding of RNA polymerase II to the *ek1* promoter was also examined by using ChIP assay. As shown in Fig 5B, 24 hours after TSA treatment, the binding of RNA polymerase II was increased significantly compared to control. Based on our results, it can be concluded that the induction of *ek1* promoter activity and endogenous expression by TSA treatment is resulted from the changes in the chromatin structure around the Sp(-40/-31) site that consequently increased the local recruitment of Sp1/Sp3 transcription factors and RNA polymerase II to the *ek1* minimal promoter region.

### TSA activation of basal *ek1* promoter activity is cell-line specific

**Fig 6:**
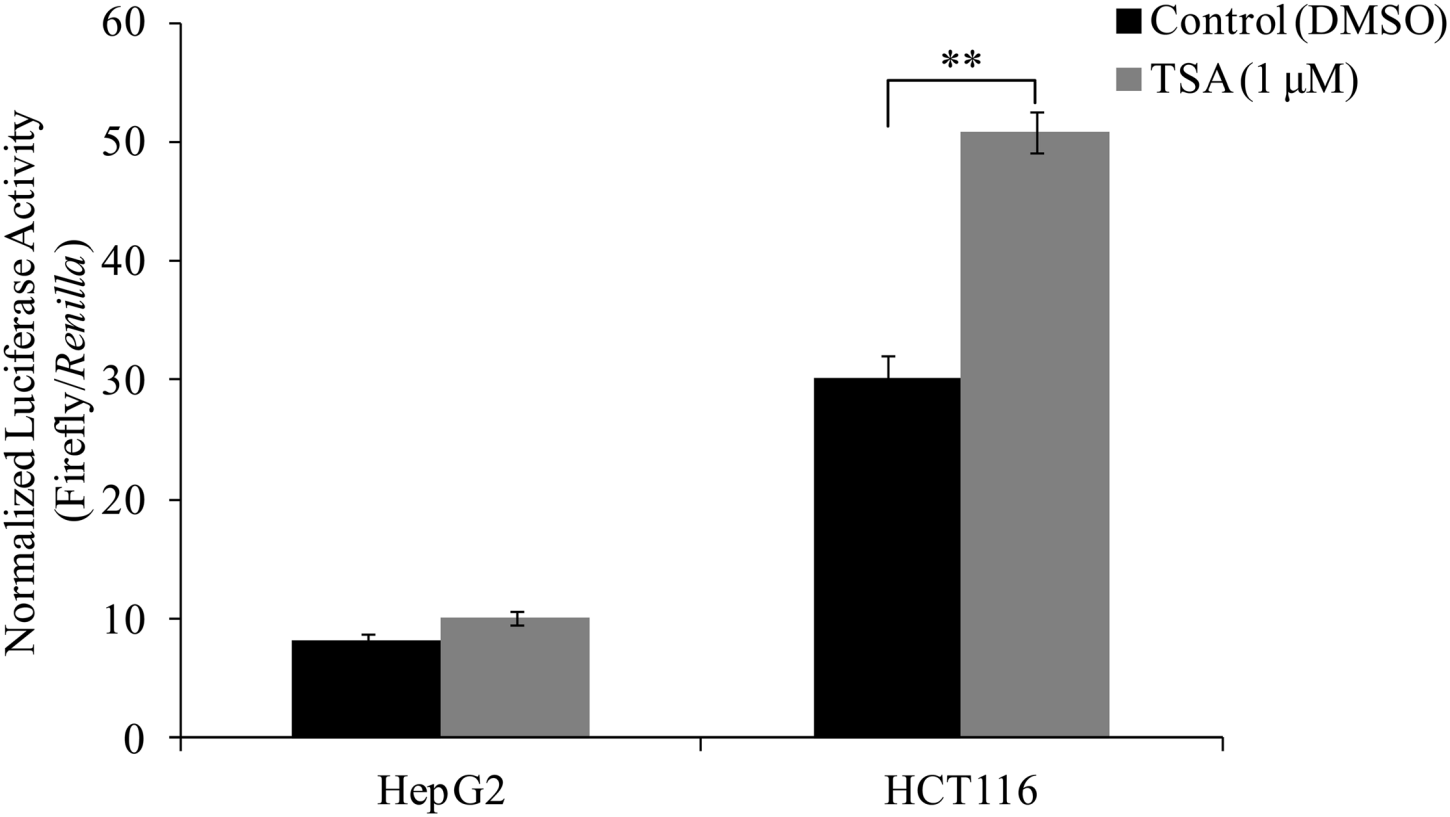
Effect of TSA on the activity of wild type *ek1* minimal promoter in HepG2 and HCT116 cells. Cells were treated with 1 μM of TSA for 24 hours (**p < 0.01 vs. DMSO control). Each bar represents the mean ± SEM of triplicate samples from three independent experiments.

According to Lykidis *et al* [3], higher levels of human *ek1* mRNA transcripts were detected in kidney, liver, heart, leukocytes, small intestine and ovary compared to the levels in colon, skeletal muscle and prostate. This suggests that *ek1* promoter might be regulated differently according to cell type. To investigate whether the TSA activation of *ek1* promoter was cell-line specific, the effect of TSA treatment on *ek1* promoter activity was studied in liver cancer cell line, HepG2. As shown in Fig 6, TSA did not significantly induce the *ek1* promoter activity as in the case of HCT116 cells.

**Fig 7:**
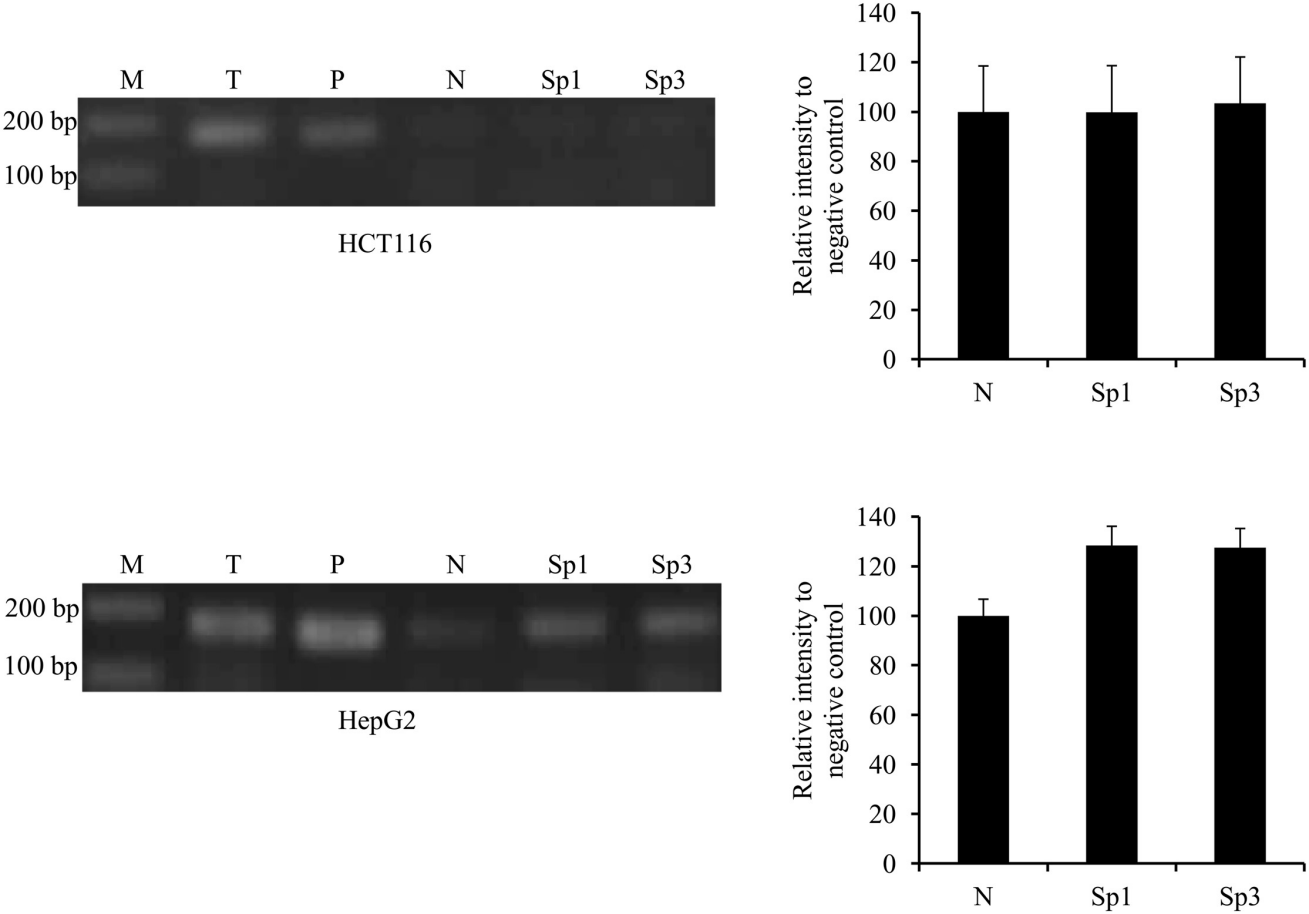
ChIP analysis of *ek1* minimal promoter region for the binding of Sp1 and Sp3 in HCT116 and HepG2 cells. PCR amplification products were resolved on 2% (w/v) agarose gel and visualized by EtBr staining. Band intensities were quantitated with Image J 1.42 and the relative intensities (compared to negative control) of PCR products from Sp1 and Sp3 immunoprecipitates were plotted. Each bar represents standard error of means (SEM) from two independent experiments. M: GeneRuler^™^ DNA Ladder Mix; T: total input sample (unprocessed chromatin); P: positive control (amplified using GAPDH Primers) and N: pre-immune normal rabbit IgG (negative control).

In order to answer the question of whether the binding of Sp transcription factors was also cell-line specific, ChIP analysis for the binding of Sp1 and Sp3 to the *ek1* minimal promoter region in HCT116 and HepG2 cells without any treatment was carried out. Fig 7 shows that PCR products were amplified from Sp1 and Sp3 antibodies precipitated DNA from HepG2 cells (lower panel) but not from HCT116 cells (upper panel). The results showed that in HepG2 cells, Sp1 and Sp3 transcription factors were already bound to the basal *ek1* promoter without TSA treatment and this explained why the promoter activity was not affected by TSA as in the case of HCT116 cells.

## Discussion

In this work, we aimed to characterize the *ek1* promoter and investigate the possibility of *ek1* gene transcription regulation by epigenetic mechanism. Based on our sequence analysis, the *ek1* gene promoter is a TATA-less, GC-rich promoter which probably belongs to the housekeeping gene family. This assumption is supported by the fact that approximately 76% of human TATA-less core promoters contain multiple Sp1 binding elements in the GC rich sequences [30] and the ubiquitous Sp1 serves as a constitutive activator of housekeeping genes by recruiting TATA-binding protein (TBP) to the promoters without recognizable consensus TATA box [31, 32].

The activity of putative *ek1* promoter (-1966/+1) was initially assessed in HepG2, HCT116 and MCF-7 cells. Under the same transfection conditions, the luciferase expression level driven by the *ek1* promoter varied with the cell line used. The transcription level of *luc2* reporter gene in the transfected promoter-luciferase reporter construct generally depends on the level of required transcription factors in the host cell. The levels of certain transcription factors are cell line dependent such as the expression level of Ets-1 in MCF-7 cell is known to be 10% lower than the other cell lines [33]. Therefore, the variation of the *ek1* promoter activities in the three cell lines tested might be due to different expression levels of the required transcription factors.

Deletion analysis revealed that the region spanning from -69 to +1 is critical for the *ek1* gene promoter basal activity. This region is CpG rich, with two putative binding sites for Sp proteins. However, only one of the Sp binding sites located at -40/-31 is critical as the positive regulatory element for *ek1* gene transcription. Sp1 and Sp3 are two closely related members of Sp-family transcription factors based on their similarities in structure and binding specificity to GC or GT motif [34, 35]. The binding of ubiquitous Sp1 to TATA-less promoters is critical for gene transcription initiation as it has been demonstrated that the transcription of TATA-less promoters requires multisubunit TFIID complex that comprises Sp1 and other coactivators like TBP and TBP-associated factors (TAFs) [36–39]. Sp1 is suggested to be involved in assembling the basal transcription machinery by interaction with the TBP/TFIID [38]. Sp1 plays an important role to tether the preinitiation complex (PIC) to TATA-less promoters by stabilizing the recruitment of TFIIB and TFIIE into PIC [32]. Since *ek1* promoter is a TATA-less promoter, the binding of Sp1 to Sp(-40/-31) site in the promoter is pivotal to the transcription initiation of *ek1* gene. Mutation introduced at this site completely abolished the *ek1* promoter activity possibly due to the disruption of PIC formation on *ek1* promoter.

Histone acetylation/deacetylation is mainly regulated by histone acetyltransferases (HATs) and histone deacetylases (HDACs) [13]. HATs acetylate the lysine residues on N-terminal tail of the histones and thus neutralizing their positive charges. This will diminish the electrostatic interactions with the negatively charged DNA and open accessibility to general transcriptional machinery, which subsequently leads to transcriptional activation. Conversely, HDACs remove the acetyl group on lysine residues, allowing more condense chromatin structure to form which causes transcriptional silencing [13]. Therefore, the balance between the activities of HDACs and HATs is important in the regulation of gene transcription. In this study, the role of histone acetylation/deacetylation in the regulation of *ek1* gene transcription was studied using HDAC inhibitor TSA. It was found that treatment of HCT116 cells with TSA caused a potent stimulation of *ek1* promoter activity. In addition, we showed that the Sp(-40/-31) site located in the GpG-enriched region of *ek1* promoter was not only essential for *ek1* basal promoter activity but also was important for TSA response. Previous studies have pointed to Sp1 and Sp3 transcription factors as the mediators of HDAC inhibition in the regulation of gene transcription [27, 40, 41]. Interestingly, the increase in *ek1* mRNA level induced by TSA is markedly lower than the TSA-mediated upregulation of *ek1* promoter activity. One possible explanation for the discrepancy between *ek1* mRNA level and its promoter activity is the involvement of other regulatory region in *ek1* promoter or post-transcriptional regulation of *ek1* gene expression.

Sp-family of transcription factors consists of four proteins designated Sp1, Sp2, Sp3 and Sp4 which are involved in the expression of a large number of housekeeping genes [26]. Sp1 and Sp3 transcription factors are highly similar in protein structure. They contain two major glutamine-rich activation domains at the N terminus and a conserved Cys2His2 zinc finger domain at the C terminus for DNA binding [34]. Due to these structural similarities, Sp1 and Sp3 share similar affinities for GC (GGGGCGGGC) or GT (GGGTGTGGC) motifs [34, 35]. Sp3 can also be directly acetylated by HAT and TSA-induced acetylation of Sp3 switches its function from the transcriptional repressor to the transcriptional activator of transforming growth factor-β receptor gene [42]. Our ChIP analysis showed that TSA induced the binding of Sp1 and Sp3 to the *ek1* promoter. We suggest that the activation of *ek1* promoter by HDAC inhibitor requires the binding of Sp1 or Sp3 to the *ek1* minimal promoter region. Previous study has shown that TSA could induce the recruitment of RNA polymerase II to the transcription factor complex for the initiation of transcription process [27]. Here, the increased binding of RNA polymerase II after TSA treatment could be due to the changes of chromatin structure or the increased binding of Sp1/Sp3 that recruits RNA polymerase II to the TSS for the activation of *ek1* transcription. Our results indicate that the activity of *ek1* minimal promoter region harboring the Sp(40/-31) site is significantly affected by histone modifications. HDACs play an important role in carcinogenesis by regulating the expression of genes involved in both cancer initiation and progression [13]. The findings from this study provide important link between chromatin modification by HDACs and *ek1* gene expression that affects cell survival.

It is worth noting that TSA up-regulation of *ek1* promoter was cell-line specific. We showed that, unlike in HCT116, the binding of Sp1 and Sp3 to *ek1* basal promoter region had already occurred in HepG2 cells before TSA treatment. The cell-type specificity of TSA effect could be due to differential regulation of *ek1* gene in different cell types as evidenced by variable level of *ek1* mRNA transcript across multiple tissues [3]. Differential regulation of *ek1* promoter was also supported by the results in this study that showed *ek1* promoter (-1966/+1) activity was clearly different among the three cell lines used.

In conclusion, this study showed that Sp(-40/-31) site is required for the transcriptional activation of the *ek1* basal promoter. The transcriptional regulation of *ek1* is associated with chromatin remodeling and Sp1/Sp3/RNA polymerase II accessibility to promoter region around the Sp(-40/-31) site. Sp1 and Sp3 not only function in concert with the basal transcriptional machinery to initiate *ek1* gene transcription, their accessibility to the target site on *ek1* promoter also depends on histone proteins modification. This study delineates the essential *cis*-acting regulatory element of *ek1* promoter and its possible modulation by chromatin structure modification that is useful for the fundamental understanding of *ek1* gene transcriptional regulation.
